# Network meta-analysis of 6 kinds of Chinese patent medicines combined with mifepristone in the treatment of uterine fibroids: A protocol for systematic review and network meta-analysis

**DOI:** 10.1097/MD.0000000000027523

**Published:** 2021-10-22

**Authors:** Chenhao Bi, Mingqi Qiao, Yuqi Jia, Haijun Wang

**Affiliations:** a College of Traditional Chinese Medicine, Shandong University of Traditional Chinese Medicine, Jinan, China; b College of Rehabilitation Medicine, Shandong University of Traditional Chinese Medicine, Jinan, China.

**Keywords:** Chinese patent medicine, mifepristone, network meta-analysis, protocol, uterine fibroids

## Abstract

**Background:**

Uterine fibroids are benign. They belong to the category of “abdominal mass” in traditional Chinese medicine, and pathogenesis is mainly caused by weakness of the body, qi stagnation, and blood stasis. Drug therapy is the preferred treatment of uterine fibroids in clinical practice, and mifepristone is the most commonly used drug. In the past decade, a large number of clinical randomized controlled trials have proven that Chinese patent medicine combined with mifepristone in the treatment of uterine fibroids has a better curative effect, fewer adverse reactions, and higher safety than mifepristone alone. However, there is a lack of evidence-based research. This study aims to integrate clinical data through network meta-analysis to provide more evidence-based medical evidence for clinical medication.

**Methods:**

The comprehensive search included Chinese and other-language databases, such as MEDLINE (PubMed), Web of Science, The Cochrane Library, China National Knowledge Infrastructure, Wanfang Data Knowledge Service Platform, China Scientific Journal Database, and China Biomedical Literature Database. Clinical randomized controlled trials of 6 Chinese patent medicines combined with mifepristone for the treatment of uterine fibroids, including Guizhi Fuling Capsule, Gongliuxiao Capsule, Gongliuqing Capsule, Danbie Capsule, Gongliuning Capsule, and Xiaojiean Capsule were retrieved. The search period was from January 2010 to April 2021. Two researchers screened the literature through EndNote and used Excel to extract data. RevMan 5.3 was used to evaluate the quality of the literature. Treatment measures were analyzed in R language, and a forest map and probability ranking map of various interventions were drawn. The network evidence map and correction comparison funnel map of various interventions were drawn by STATA 14.0 software.

**Results:**

This study provides the clinical efficacy and safety of network meta-analysis of 6 kinds of Chinese patent medicines combined with mifepristone in the treatment of uterine fibroids will be systematically evaluated or descriptively analyzed.

**Conclusion:**

This study's purpose is to provide a reference for the clinical treatment of uterine fibroids to choose more effective intervention therapies.

## 1 Introduction

Uterine fibroids are benign tumors growing in the myometrium, mucous membrane, or surface of the uterus. Uterine fibroids belong to the category of “abdominal mass” in traditional Chinese medicine, and pathogenesis is mainly caused by weakness of the body, qi stagnation and, blood stasis. Uterine fibroids occur mostly in women aged 30 to 50 years old, and the incidence is as high as 20% to 40%. The symptoms are mild and difficult to detect in the early stage, or there may be no symptoms. However, with the development of fibroids, patients may have menstrual abnormalities, abdominal pain, and even infertility, which has a certain impact on their physical and mental health.^[1]^ Studies have shown that the growth of uterine fibroids is related to many factors. Vascular endothelial growth factor (VEGF), an angiogenesis-promoting factor that plays an important role in the generation and development of tumor blood vessels, is one of the important indicators for the treatment and prognosis of various types of cancer. Expression of VEGF in uterine fibroids is significantly higher than that in surrounding tissues.^[2–3]^ Matrix metalloprotein 9 (MMP-9) regulates the repair and injury of uterine smooth muscle cells by promoting the metabolism and decomposition of collagen fibers and promotes interstitial decomposition of uterine smooth muscle cells to induce the formation of new blood vessels, thereby promoting the differentiation or proliferation of smooth muscle cells.^[4–5]^ The mitogen-activated protein kinase pathway regulates matrix metalloproteinase 2 (MMP-2) protease, and an increase in MMP-2 will destroy links between cells, enhancing the invasion and migration abilities of uterine leiomyoma cells.^[6]^

To avoid the impact of surgery on fertility, drug therapy is the preferred treatment of uterine fibroids in clinical practice, and mifepristone is the most commonly used drug. Mifepristone antagonizes progesterone activity and promotes decidual cell necrosis and uterine fibroid atrophy. However, since mifepristone is a hormone drug, there are many adverse reactions with high-dose administration, and the curative effect is not very stable; overall, it is not suitable for long-term single-drug use.^[7–8]^

In the past decade, a large number of clinical randomized controlled trials (RCTs) have proven that Chinese medicine plays an irreplaceable role in the treatment of uterine fibroids. Compared with mifepristone alone, Chinese patent medicine combined with mifepristone for the treatment of uterine fibroids has a better curative effect and higher safety. Guizhi Fuling Capsule can reduce the transcription and protein expression of downstream genes by inhibiting the mitogen-activated protein kinase pathway, suppressing the proliferation, migration, and invasion of uterine fibroid cells. Guizhi Fuling Capsule also promotes immunity by increasing levels of CD3+ and CD4+/CD8+, thereby improving the therapeutic effect on uterine fibroids.^[9]^ By reducing leptin and prolactin levels and downregulating activity of the Rac1/MMP-2 pathway, Gongliuxiao Capsule inhibits the growth of uterine leiomyoma cells or promotes apoptosis.^[10]^ Gongliuqing Capsule inhibits uterine leiomyoma cell proliferation and induces apoptosis,^[11]^ and Danbie Capsule inhibits the proliferation and metastasis of leiomyoma cells by regulating MMP-9 and VEGF levels.^[12]^ Gongliuning Capsule reduces serum MMP-9 and VEGF expression.^[13]^ Xiaojiean Capsule significantly decreases the volume of uterine fibroids and pain.^[14]^

At present, studies in this area are mostly RCTs to verify the clinical efficacy of a single Chinese patent medicine therapy on uterine fibroids, whereas there is no evidence-based evaluation that compares the clinical efficacy of 6 Chinese patent medicine therapies for uterine fibroids at the same time. Therefore, this study investigated 6 Chinese patent medicine therapies commonly used in clinical practice and used network meta-analysis (NMA) to integrate relevant clinical evidence. After summarizing different interventions in the same body of evidence, a quantitative comprehensive statistical analysis was performed to compare the clinical efficacy of the 6 different Chinese patent medicine therapies for the treatment of uterine fibroids. Our purpose is to provide a reference for the clinical treatment of uterine fibroids to choose more effective intervention therapies.

## 2 Methods and analysis

### 2.1 Study registration

The protocol and registration information are available at PROSPERO (https://www.crd.york.ac.uk/prospero/display_record.php?ID=CRD42021258868, registration number: CRD42021258868). We performed this meta-analysis according to Preferred Reporting Items for Systematic review and Meta-Analysis Protocols (PRISMA-P) statement.^[15]^

### 2.2 Inclusion criteria for study selection

#### 2.2.1 Types of studies

We included only published clinical RCTs in English and Chinese.

#### 2.2.2 Types of participants

The patients were all clinically diagnosed with uterine fibroids. The age, sex, race, and region of the patients were not limited. The diagnostic criteria used for uterine fibroids complied with “Obstetrics and Gynecology”,^[16]^ “Practice of Obstetrics and Gynecology”,^[17]^ “Chinese expert consensus on the diagnosis and treatment of uterine fibroids”,^[18]^ “Gynecology of Traditional Chinese Medicine”,^[19]^ and “Guidance Principle of Clinical Study on New Drug of Traditional Herbal Medicine”.^[20]^

#### 2.2.3 Types of interventions and comparators

The treatment group was treated with 1 of the 6 Chinese patent medicines (Guizhi Fuling Capsule, Gongliuxiao Capsule, Gongliuqing Capsule, Danbie Capsule, Gongliuning Capsule, and Xiaojiean Capsule) combined with mifepristone. The control group was treated with mifepristone alone. The batch, dose, administration times, and course of treatment in the 2 groups were not limited.

#### 2.2.4 Types of outcome measures

##### 2.2.4.1 Primary outcomes

Total effective rates, total effective rates = (total cases – invalid cases)/total cases × 100%; Uterine fibroid volume.

##### 2.2.4.2 Secondary outcomes

Hormone index, including progesterone, oestradiol, follicle-stimulating hormone, and luteinizing hormone; and adverse reaction rate.

#### 2.2.5 Exclusion criteria

Other diseases; repeated published study; incomplete or wrong data; no clear diagnostic criteria; no clear criteria for efficacy evaluation; no clear dosage or dosage form; and no definite course of treatment.

### 2.3 Search methods for identification of studies

We conducted a systematic search of the following electronic databases from January 2010 to April 2021: MEDLINE (PubMed), Web of Science, The Cochrane Library, China National Knowledge Infrastructure, Wanfang Data Knowledge Service Platform, China Scientific Journal Database, and China Biomedical Literature Database. The following search terms were used: Uterine Fibroids, Guizhi Fuling Capsule, Gongliuxiao Capsule, Gongliuqing Capsule, Danbie Capsule, Gongliuning Capsule, Xiaojiean Capsule, Mifepristone, Random, etc. Equivalent search words were used in the Chinese databases. According to the search modes of different databases, keywords were combined with free words for a comprehensive search. A search strategy created according to the Cochrane handbook guidelines will be conducted in all electronic databases.^[21]^ The search strategy for PubMed is shown in Table 1. This search strategy was modified as required for other electronic databases.

**Table 1:** Search strategy used in the PubMed database.

Number	Search terms
1	Search “Uterine Fibroids”
2	Search (((((((((((((((((((Leiomyomas) OR Fibroid Tumor) OR Fibroid Tumors) OR Tumor, Fibroid) OR Tumors, Fibroid) OR Fibromyoma) OR Fibromyomas) OR Fibroid) OR Fibroids) OR Fibroid Uterus) OR Uterus, Fibroid) OR Fibroma, Uterine) OR Fibromas, Uterine) OR Uterine Fibroma) OR Uterine Fibromas) OR Fibroids, Uterine) OR Fibroid, Uterine) OR Uterine Fibroid) OR Uterine Fibroids) OR Leiomyoma, Uterine
3	#1 AND #2
4	Search “Guizhi Fuling Capsule”
5	Search “Gongliuxiao Capsule”
6	Search “Gongliuqing Capsule”
7	Search “Danbie Capsule”
8	Search “Gongliuning Capsule”
9	Search “Xiaojiean Capsule”
10	#4 OR #5 OR #6 OR #7 OR #8 OR #9
11	Search “Mifepristone”
12	#10 OR #11
13	Search “Randomized Controlled Trial” [Publication Type]
14	Search (((((Controlled Clinical Trial[Title/Abstract]) OR Randomized[Title/Abstract]) OR Placebo[Title/Abstract]) OR Randomly[Title/Abstract]) OR Trial[Title/Abstract]) OR Groups[Title/Abstract]
15	#13 OR #14
16	#3 AND #12 AND #15

### 2.4 Data collection and analysis

#### 2.4.1 Selection of studies

**Figure 1:**
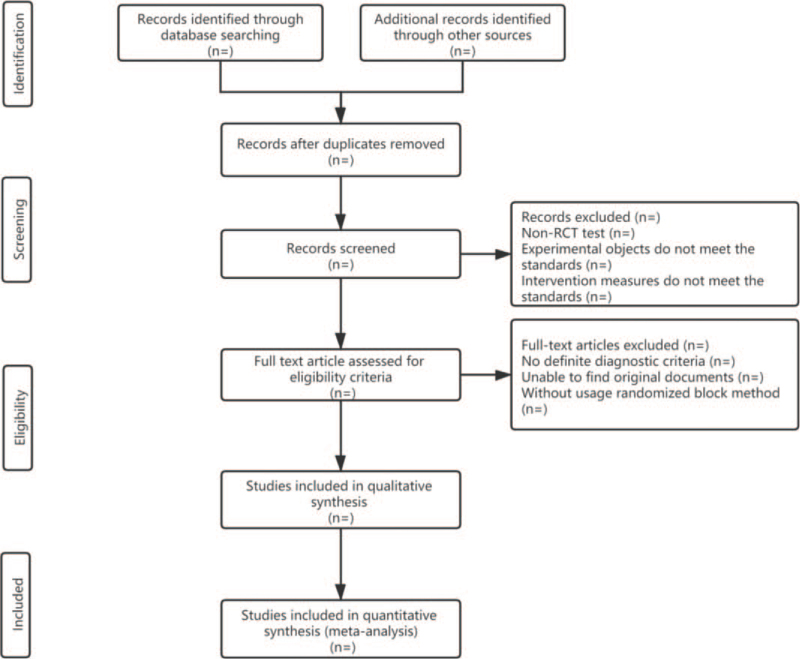
PRISMA flow diagram. PRISMA =  Preferred Reporting Items for Systematic review and Meta-Analysis.

According to the abovementioned electronic database search strategy, 2 researchers searched Chinese and English electronic databases. Researchers used Endnote X7 software (Thomson Corporation is based in Stanford, Connecticut) to independently check the title and summary of the retrieval results, delete duplicate literature, select appropriate literature to download the full text, and present the data according to the predetermined table. When disagreement occurred, third-party experts were invited to discuss and research and finally make a decision. The PRISMA flowchart selected for this process is shown in Figure 1.^[22]^

#### 2.4.2 Data extraction and management

Two researchers extracted data independently. The data extraction content included the basic information of the included literature (including the first author, published journal and year, and study title); relevant information of the experimental group and the control group (including the number of cases, disease course, age, intervention measures, treatment course, and outcome indicators); and the design type and the quality evaluation information (e.g., random method, blind method, allocation concealment, completeness of outcome data, selective reporting results, and other sources of bias). The original authors were contacted in cases of missing data. When there was disagreement regarding data extraction, a third reviewer was consulted for consensus.

#### 2.4.3 Assessment of risk of bias in included studies

Risk of bias was assessed by 2 researchers using the Cochrane risk of bias tool recommended by the Cochrane reviewer's handbook. According to the evaluation content of the manual, each included study was evaluated from random methods, allocation concealment, blinding, completeness of outcome data, selective reporting results, and other sources of bias. Each project was divided into 3 types of results: high risk, low risk, and uncertainty risk.^[23–24]^

### 2.5 Data synthesis and statistical methods

RevMan 5.3 (Cochrane UK, Summertown Pavilion, 18 - 24 Middle Way, Oxford, UK) was used to evaluate the quality of the literature data, and R language (Oakland University, New Zealand) was used to analyze treatment measures. A forest map and probability ranking map of various interventions were drawn. The network evidence map and correction comparison funnel map of various interventions were drawn with STATA 14.0 software (Lakeway Drive, College Station, Texas, USA). The R language program netmeta was used through relevant instructions with the Bayesian Markov Chain Monte Carlo algorithm for random effect model data results to achieve network data analysis and mapping. The odds ratio and its 95% confidence interval were used to represent the effect amount in the enumeration data. The measurement data used the mean difference and its 95% confidence interval to represent the effect. When the data were incomplete, the author was contacted; if we were unable to obtain complete data, the article was excluded. The ranking probability diagram was used to rank the efficacy of interventions, with α = 0.05 as the test level.

### 2.6 Assessment of heterogeneity

Heterogeneity was assessed by Cochrane analysis. The I^2^ index was used for statistical heterogeneity assessment and x^2^ for subgroup analysis based on heterogeneity factors. The clinical and methodological heterogeneity of the included studies was evaluated, and levels of fit of the fixed-effect model and the random-effect model were compared. When there was no heterogeneity among studies (*P* ≥ .1, I^2^ ≤ 50%), a fixed-effect model combined with effect quantity analysis was used. When there was large heterogeneity (*P* < .1, I^2^ > 50%) between the studies, a random-effect model was used combined with effect quantity analysis, and the source of heterogeneity was analyzed. After sensitivity analysis or subgroup analysis according to its source, descriptive analysis was carried out when impossible to determine the source.^[25]^

### 2.7 Subgroup and sensitivity analyses

There was no subgroup plan. If significant heterogeneity was detected and data were sufficient, we used subgroup analysis to determine the reasons for the heterogeneity and compare the effects of each group.

### 2.8 Publication bias

If at least 10 studies comparing the same group of treatments were included, and we assessed risk of publication bias using funnel plots, as recommended in Cochrane Handbook. A graph showing inverted funnel-like symmetry indicated that the possibility of publication bias was relatively small. A funnel chart that was asymmetric or incomplete indicated a greater possibility of publication bias.

### 2.9 Quality of evidence

Two researchers will independently appraise the strength of the evidence in compliance with Grades of Recommendation, Assessment, Development, and Evaluation guidelines. Considerations of evidence quality assessment include study limitation, consistency of effect, imprecision, indirectness, and publication bias. The evidence quality was classified into 4 levels (high, medium, low, and very low).^[26]^

### 2.10 Ethics and dissemination

The NMA protocol was approved by the local institutional review board and ethics committee; it does not involve private information, nor does it require further ethical approval or informed consent.

## 3 Discussion

Compared with Western medicine, traditional Chinese medicine has unique advantages in the treatment of uterine fibroids.^[27]^ The treatment is to promote qi and activate blood, disperse stagnation, and break stasis, following the principle of eliminating disease without hurting health. Traditional Chinese medicine can both improve efficacy and reduce side effects.

Most studies to date have only reported the efficacy of a single Chinese patent medicine for the treatment of uterine fibroids, and comparative studies with NMA are lacking. Therefore, the purpose of this study was to use a high-quality system to evaluate 6 commonly used Chinese patent medicine therapies, to use the NMA method to analyze effective rates, uterine fibroid volume, hormone index, and other indicators and to evaluate the quality of the curative effect of the 6 Chinese patent medicine therapies. To determine the effects of 6 Chinese patent medicine therapies in the treatment of uterine fibroids, we sorted them according to the pros and cons of index effects. Then, we screened out the best evidence of clinical treatment measures and used the analytic hierarchy process to evaluate the quality of the evidence. Our review may provide the best possible Chinese patent medicine therapy options and reliable evidence-based medicine for the clinical treatment of uterine fibroids and, to a certain extent, provide new insight into the auxiliary treatment of uterine fibroids by Chinese patent medicine therapy.

## Author contributions

**Conceptualization:** Chenhao Bi, Haijun Wang.

**Data curation:** Chenhao Bi, Yuqi Jia.

**Formal analysis:** Chenhao Bi, Yuqi Jia.

**Funding acquisition:** Haijun Wang, Mingqi Qiao.

**Investigation:** Chenhao Bi, Yuqi Jia.

**Methodology:** Chenhao Bi, Yuqi Jia.

**Project administration:** Chenhao Bi.

**Resources:** Chenhao Bi, Yuqi Jia.

**Software:** Chenhao Bi, Yuqi Jia.

**Supervision:** Yuqi Jia, Haijun Wang, Mingqi Qiao.

**Validation:** Chenhao Bi, Yuqi Jia, Haijun Wang, Mingqi Qiao.

**Visualization:** Chenhao Bi, Yuqi Jia.

**Writing – original draft:** Chenhao Bi.

**Writing – review & editing:** Yuqi Jia, Haijun Wang, Mingqi Qiao.
